# Biodegradable poly(ethylene glycol-glycerol-itaconate-sebacate) copolyester elastomer with significantly reinforced mechanical properties by in-situ construction of bacterial cellulose interpenetrating network

**DOI:** 10.1038/s41598-024-56534-z

**Published:** 2024-03-26

**Authors:** Lisheng Tang, Yuanyuan Jin, Xiaoyan He, Ran Huang

**Affiliations:** 1 Academy for Engineering and Applied Technology; Yiwu Research Institute; Zhuhai Fudan Innovation Institute, Fudan University, Shanghai, 200433 China; 2 Center for Innovation and Entrepreneurship, Taizhou Institute of Zhejiang University, Taizhou, 318000 Zhejiang China; 3 Department of Polymer Science and Engineering, Zhejiang University, Hangzhou, 310027 Zhejiang China

**Keywords:** Biodegradable, Copolyester elastomer, PEGIS, Bacterial cellulose, Interpenetrating network, Biomaterials, Soft materials

## Abstract

To address the concern that biodegradable elastomers are environmental-friendly but usually associated with poor properties for practical utilization, we report a star-crosslinked poly(ethylene glycol-glycerol-itaconate-sebacate) (PEGIS) elastomer synthesized by esterification, polycondensation and UV curing, and reinforced by bacterial cellulose (BC). The interpenetrating network of primary BC backbone and vulcanized elastomer is achieved by the “in-situ secondary network construction” strategy. With the well dispersion of BC without agglomeration, the mechanical properties of PEGIS are significantly enhanced in tensile strength, Young’s modulus and elongation at break. The reinforcement strategy is demonstrated to be efficient and offers a route to the development of biodegradable elastomers for a variety of applications in the future.

## Introduction

An elastomer is a type of polymer that exhibits a low modulus and high elasticity, with the ability to rapidly return to its original state after the removal of external force. Due to its distinctive qualities, elastomers play an indispensable role in both the economy and daily life^1–4^. To meet the increasing performance requirements, continuous efforts have been dedicated on elastomers for better properties in strength, wear resistance, stability and aging resistance. On the other hand, these excellences cause the waste products of traditional elastomers more difficult to dispose of, resulting in long-term environmental pollution^5,6^. Meanwhile, most elastomers come from non-renewable fossil resources with poor sustainability. In recent years, due to the huge pressure of carbon emissions, the development of new elastomers that do not rely on fossil resources has become a strong demand. Bio-based elastomers can be obtained directly from natural plants or synthesized by bio-based monomers derived from biomass resources, making them an ideal option for resource conservation and carbon reduction in the elastomer industry.

Aliphatic polyester has good biodegradability and becomes a research hotspot for environmental-friendly materials. In the field of plastic, linear polyesters, such as poly(lactic acid) (PLA), polyglycolide acid (PGA), polycaprolactone (PCL) and their copolymers, have been intensively researched for decades, and their industrial scale are growing rapidly^7–9^. Inspired by these linear polymer, polyesters with three-dimensional network have also attracted more and more attention^10,11^. Similar to vulcanized rubber, the cross-linked network is introduced by “star” monomers to construct branched chains, giving material the ability to quickly recover the shape under large deformation. Poly (glycerol sebacate) (PGS), polyoctyl citrate (POC) and their copolymers are main biodegradable polyester elastomers that have been extensively studied. As early as 2002, Langer et al. synthesized PGS for the first time, which is an in-situ cross-linked polyester elastomer with three-dimensional network structure and excellent resilience^10,12^. In 2011, Zhang et al.^13^ developed the first chemically crosslinked polyester elastomer. Afterwards, more novel bio-based elastomers have been developed^14,15^. 

Biodegradable polyester elastomers are expected to replace traditional elastomers with a limited-service life, with the promoting benefits on environmental protection, low-carbon, and sustainability, and provide more potential usage for fields such as biomedicine and daily necessities. However, due to the easy hydrolysis and space hindrance of ester linkage, polyester always encounters the problem of weak mechanical properties and thermal stability, which is especially concerned in rubber industry, thereafter, developing new high-performance biodegradable polyester elastomers and further reinforcement to make them practically useful is a continuous effort to realize their natural environmental-friendly and low-carbon benefits. It was reported that introducing appropriate rigid structural crosslinkers and increasing the crosslink density of polyester elastomers can improve the mechanical properties^16–18^. However, chemical modification processes are complex, technically challenging, and relatively expensive. Blending with nanofillers is a simple and effective physical modification to enhance the mechanical properties of polyester elastomers. Liu et al.^19^ prepared a series of nano-silica (nano-SiO_2_)/ poly(glycerol-sebacate-citrate) (PGSC) biodegradable elastomers using a solvent-assisted in-situ dispersion method. With the 16.67 wt% addition of nano-SiO_2_, the tensile strength and modulus of the PGSC increased by 602.20% and 258.21%, respectively. Yan et al.^20^ prepared MWCNT/PGS nanocomposites with varying filler loadings via solution mixing, the addition of fillers increased the overall mechanical stiffness without significantly compromising elasticity. Biomass materials, being renewable, biodegradable, abundant in source, and high in production volume, have also emerged as reinforcing fillers that have garnered significant attention in recent years^21–23^. Among biomass fillers, cellulose, as the most abundant and widely distributed natural polymer, is regarded as one of the most attractive and promising reinforcement fillers. The tensile strength of cellulose nanocrystal/PGS composite material obtained by blending and solvent casting was more than doubled comparing to pure PGS^24^.

Bacterial cellulose (BC) is an extracellular polysaccharide produced by microbial fermentation, sharing the same chemical structure as plant cellulose. But unlike plant cellulose, BC is devoid of other polysaccharides such as lignin and hemicellulose, which simplifies the purification process. Controlled cultivation allows the direct production of uniform BC dispersions, while it has always been a challenge of cellulose dispersion. Furthermore, BC exhibits higher chemical reactivity and accessibility than plant cellulose, making it more conducive to chemical modifications, grafting reactions, and cross-linking reactions. As one of the most promising cellulose materials currently available, BC holds vast potential in the field of reinforcement for biodegradable materials. Maryam et al.^25^ prepared micro-nano-sized bacterial cellulose and incorporated it as a filler into bioplastics, resulting in an impressive enhancement of both tensile strength and tensile modulus of the bioplastics. Cai et al.^26^ prepared a biocompatible PHB/BC nanocomposite scaffold by impregnating BC nanofibers into PHB matrix, resulting in a material with micro and nano-porous structures. The PHB/BC nanocomposite exhibited excellent mechanical properties and improved biocompatibility comparing to the pure PHB.

Nevertheless, the uniform dispersion of BC with an ultra-high aspect ratio in a polymer matrix remains a challenge: the original BC dispersion itself holds a 3D net structure, which is difficult to be embedded into the polymer matrix; on the other hand, if being reduced to micro-nano dimensions, it loses the advantage of exceptionally high aspect ratio. This difficulty is even more challenging in elastomers modification, where it has its own cross-linked network. It is a reasonable guess that the problem of interpenetrating network is why BC-reinforced polyester elastomer has not been reported to the best of authors’ knowledge.

**Figure 1:**
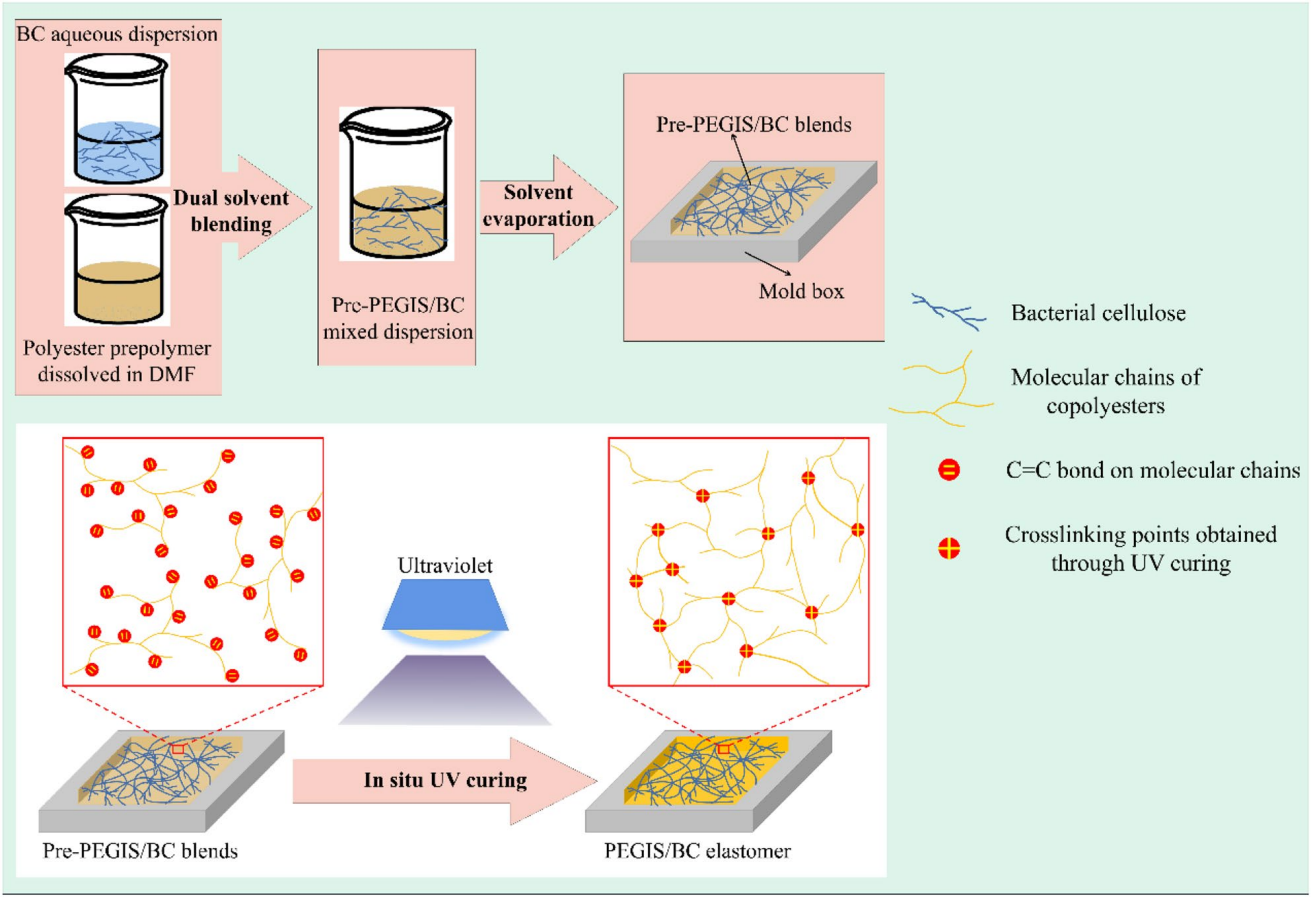
The scheme of “in-situ secondary network construction”.

**Figure 2:**
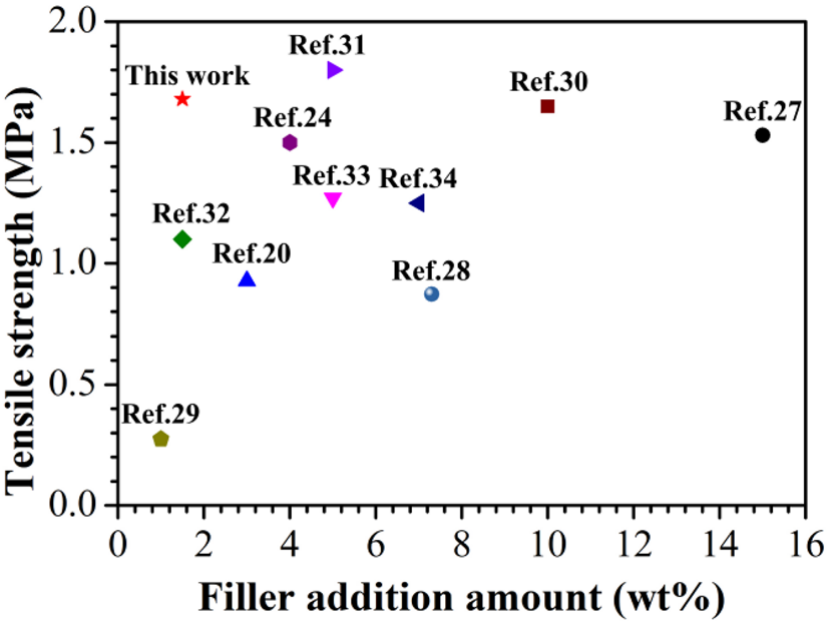
Comparison of the mechanical properties of the elastomer prepared in this work with similar elastomers reported in previous literature (Refs.^20,24,27–34^).

In this work, poly(ethylene glycol-glycerol-itaconate-sebacate) (PEGIS) copolyester elastomer is synthesized and reinforced by BC. The key technique to overcome the above concern involves the uniform mixing of unsaturated polyester precursors (pre-PEGIS) with BC through a co-solvent blending process, then well-dispersed BC/linear polyester blends is obtained through solution casting, and a final UV curing vulcanized the linear chains to construct a secondary network interpenetrating the backbone BC network. The scheme of this “in-situ secondary network construction” to keep the BC in its natural relaxed state to effectively reinforce the elastomer is illustrated in Fig. 1. The chemical microstructure, morphology, thermal and mechanical properties, and biodegradability of the composite are characterized. PEGIS copolyester elastomers are synthesized utilizing bio-based monomers derived from renewable resources. Compared to petroleum-based polymers, using bio-based sources reduces reliance on non-renewable resources, hence improving sustainability. The copolyester elastomers exhibit commendable biodegradability, aiding in alleviating stress on land and water resources while mitigating the enduring impact of waste. The elastomers are cured by UV irradiation, facilitating rapid solidification without the requirement for conventional thermal curing methods. This not only diminishes production time and energy consumption but also enhances manufacturing efficiency. The BC, characterized by exceptionally high strength and a substantial aspect ratio, is served as an effective reinforcing filler for the copolyester elastomers. And through the co-solvent blending process, BC is uniformly dispersed into the elastic matrix. Even little amounts of BC can significantly enhance the mechanical properties of PEGIS/BC. Compared to previous reports, the copolyester elastomer prepared in this work can achieve relatively high mechanical strength with little filler addition as shown in Fig. 2.

## Experimental

### Reagents and materials

Itaconic acid (≥ 99.7%) was purchased from Zhejiang Guoguang Biochemical Co., Ltd. 2-Hydroxy-4′-(2-hydroxyethoxy)-2-methylpropiophenone (HHMP, ≥ 98.0%), sebacic acid (≥ 99%) and stannous chloride (SnCl_2_, ≥ 99%) were purchased from Aladdin Reagent Co., Ltd. Glycerol (AR grade) and PEG with Mn ≈ 200 g/mol were purchased from Shanghai Macklin Biochemical Co., Ltd. Bacterial cellulose (BC) aqueous dispersion with mass fraction of 0.65% was purchased from Guilin Qihong Technology Co., Ltd. The diameter and length of BC are 50–100 nm and 20 μm, respectively. Ethanol (≥ 99.7%) and hydroquinone (≥ 98.0%) were purchased from Shanghai Chemical Reagent General Factory. *N*, *N*-Dimethylformamide (DMF, ≥ 99.5%) was purchased from Shanghai Lingfeng Chemical Reagent Co., Ltd. Deionized water was used in the whole process (Fig. 1).

### Synthesis of the polyester prepolymer

Itaconic acid (0.3 mol), sebacic acid (0.7 mol), PEG200 (0.3 mol), and glycerol (0.7 mol) were charged into a 500 mL three-necked round-bottom reactor equipped with a mechanical stirrer and N_2_ inlet in the presence of SnCl_2_ (0.5 wt% of total reactants) and hydroquinone (0.2 wt% of total reactants) as the catalyst and inhibitor, respectively. The esterification reaction was conducted at 130 °C for 3 h under N_2_ atmosphere. And then the reaction temperature was increased to 160 °C to carry out the melt polycondensation reaction for 5 h under N_2_ atmosphere. After that, the polyester prepolymer was dissolved by ethanol, flocculated with water, and washed repeatedly for 3 times to remove impurities. Most of the solvents in the flocculated polyester prepolymer were removed by rotary evaporator. Finally, the polyester prepolymer was dried in a vacuum oven at 80 °C for at least 6 h for future use, named as pre-PEGIS. The yield of the pre-PEGIS was calculated to be 80.78%.

### Preparation of the PEGIS

Three sets of pre-PEGIS were weighed and added different amounts of BC aqueous solution so that the mass fraction of BC to pre-PEGIS was 5 wt‰, 10 wt‰, 15 wt‰, respectively. According to the ratio of water to DMF of 1:2, DMF was add to the mixture, so that the DMF/AQUA mixture could dissolve the polyester prepolymer. Then, a photoinitiator (HHMP) equivalent to 0.5 wt% of the mass of the pre-PEGIS was added respectively. The mixture was stirred magnetically at 60 °C for 2 h, and then transferred to the rotary evaporator to completely remove the solvent. The obtained three sets of photoinitiator intermediates were molded to in a certain shape, and then cured under Ultraviolet (UV) irradiation (365 nm) for 30 min, to obtain three sets of degradable PEGIS elastomers. Pure polyester samples were also prepared as the reference in the same methods. According to the content of bacterial cellulose, the samples were marked as PEGIS, PEGIS/BC5, PEGIS/BC10 and PEGIS/BC15.

### Characterization

Gel Permeation Chromatography (GPC) analysis was carried out to determine the number-average molecular weight (M_n_), weight-average molecular weight (M_w_) and poly dispersity index (PDI) using a PL-GPC220 Gel Permeation Chromatograph (Agilent Technologies, USA). Tetrahydrofuran was used as the eluent at a flow rate of 1 mL/min. The FTIR were characterized on Nicolet iS 10 (Thermo Fisher Scientific, USA) with the scanning resolution of 4 cm^−1^ over the wavelength range from 4000 to 400 cm^−1^ and 2000 to 400 cm^−1^. Proton (^1^H) Nuclear Magnetic Resonance (NMR) spectra were measured at room temperature on a 600 MHz Bruker AV-600 spectrometer in methyl sulfoxide. The rheological measurements were taken on an AR2000EX dynamic rheometer (TA instruments, USA) with 25 mm diameter parallel-plate geometry at 25 °C. Dynamic oscillatory shear measurements were performed with the frequency range of 0.01–100 Hz and strain of 1%, respectively. Mechanical properties were measured on a PT-1166Z Universal Testing Machine (Dongguan perfect-group Instrument, China) at a crosshead speed of 50 mm/min at room temperature. The size of the dumbbell spline is 25 mm in length, 4 mm in width and 2 mm in thickness. The surface morphology of BC and the section morphology of PEGIS and PEGIS/BC were observed by scanning electron microscopy (SEM; S4800, Hitachi, Japan). Differential scanning calorimetry (DSC) analysis was performed on DSC-3 (Mettler Toledo, Switzerland) under a nitrogen atmosphere. About 8 mg of the samples were placed into alumina crucibles and heated to 150 °C at a heating rate of 10 °C/min and kept for 2 min to eliminate previous thermal history, followed by cooling down to − 60 °C and finally heating to 150 °C at a cooling/ heating rate of 10 °C/min. The biodegradability of the PEGIS and PEGIS/BC were characterized by composting according the EN ISO standard^35^. The degree of crosslinking can be characterized by analyzing the content of gel (crosslinking) and sol (non-crosslinking) in the copolyester elastomer PEGIG and PEGIS/BCs. The original weight (W_o_) of PEGIG or PEGIS/BCs was weighed, and then the sample was immersed in tetrahydrofuran for 24 h. The swollen sample was dried overnight and the final weight (W_f_) was measured. The sol and gel content are calculated using the following equation.

$$Sol\mathrm{ \%}= \frac{{W}_{o}-{W}_{f}}{{W}_{f}}\times 100\mathrm{\%}$$

$$Gel\mathrm{ \%}=100\mathrm{\%}-Sol\mathrm{ \%}$$

## Results and discussion

### Chemical structure

**Figure 3:**
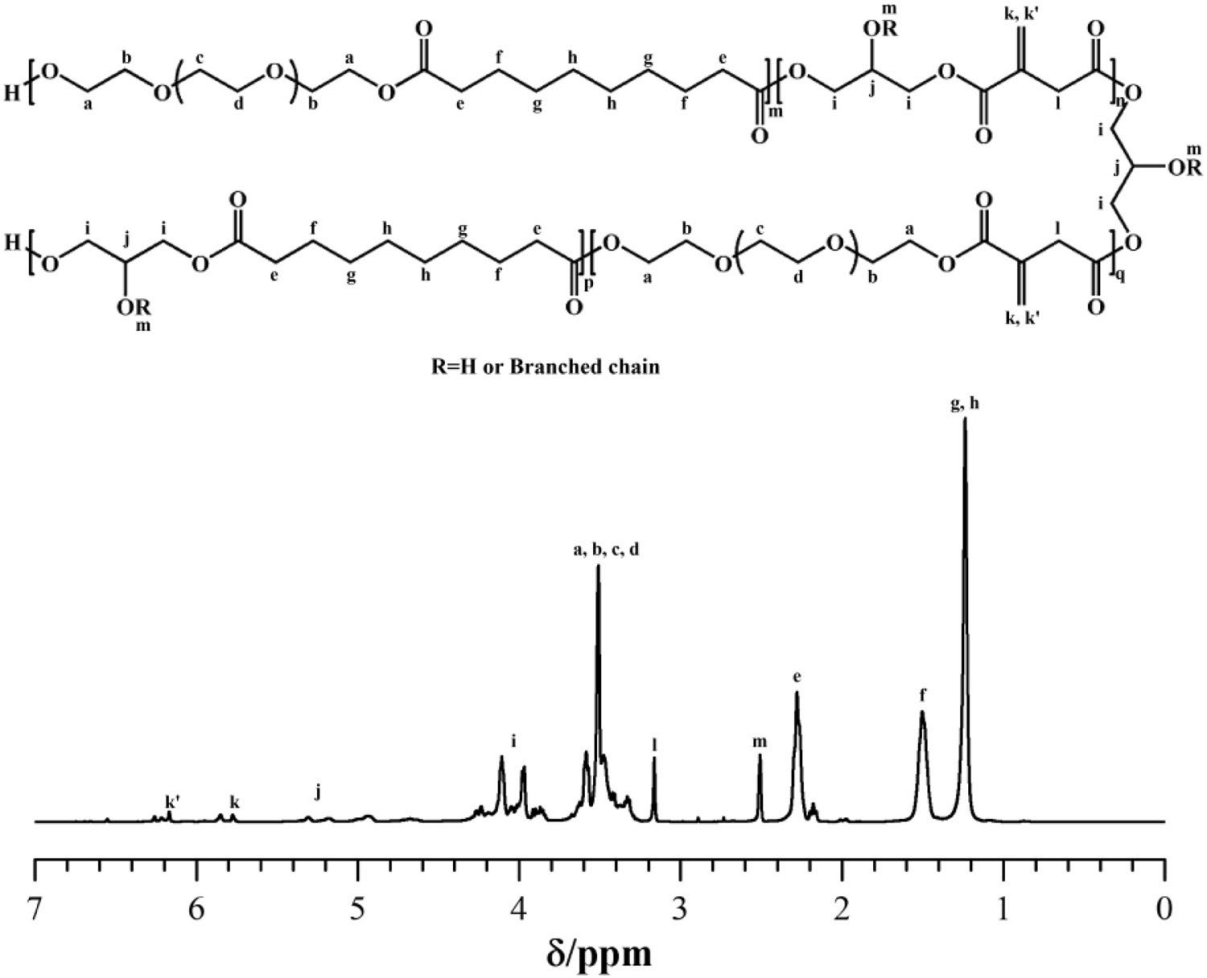
^1^H NMR spectrum of pre-PEGIS.

The pre-PEGIS was characterized by GPC and NMR to determine the chemical structure. The molecular weight M_n_ and M_w_ of the pre-PEGIS are 2078 g/mol and 4340 g/mol, and the polymer dispersion index PDI = 2.088. The ^1^H NMR spectrum of pre-PEGIS is shown in Fig. 3, typical signals can be observed at 2.28 ppm, 1.50 ppm and 1.24 ppm corresponding to the H_e_, H_f_, H_g_ and H_h_ of the sebacic acid-based units. The peaks between 3.75 and 4.40 ppm represent bonds from PEG units. Moreover, the peaks between 5.75 and 6.30 ppm illustrate that the pendent double bonds, –C(=CH_2_)–, are well kept after the polycondensation as a result of the inhibition effect of hydroquinone. The results indicate that pre-PEGIS was successfully synthesized by esterification and polycondensation of sebacic acid, itaconic acid, PEG and glycerol. According to the results of ^1^H NMR spectrum (Fig. 3), the molar fractions of each unit were calculated and listed in Table 1. The molar content of carbon–carbon double bond available shall be the same as that of the itaconic acid unit, which is 9.53 mol%.

**Table 1:** Mole fraction of each unit in the pre-PEGIS.

	Mole fraction (mol%)
Sebacic acid units	34.04
Itaconic acid units	9.53
Glycerol units	38.81
PEG200 units	17.62

**Figure 4:**
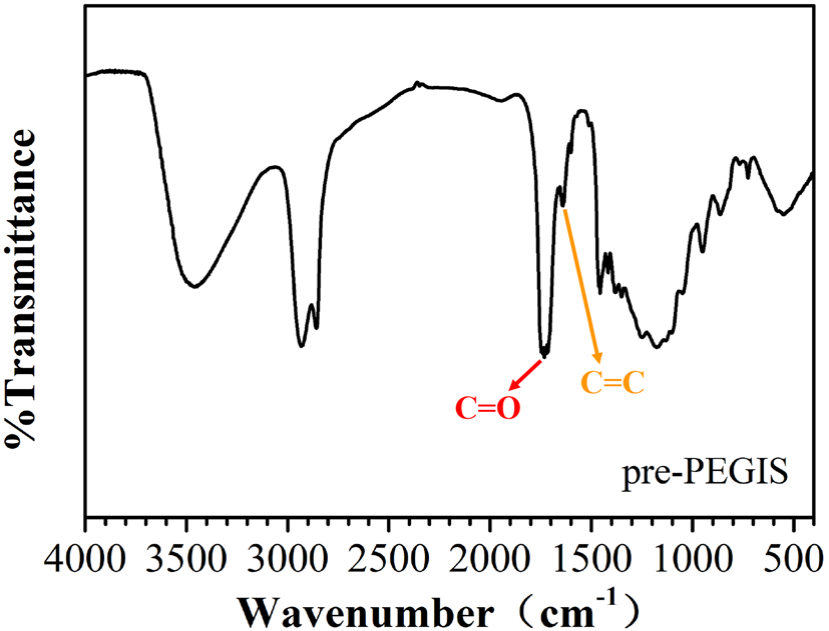
FTIR spectrum of pre-PEGIS.

In addition, the FTIR spectrum of pre-PEGIS presents characteristic peaks of ester carbonyl and carbon–carbon double bond at 1732 cm^−1^ and 1650 cm^−1^ respectively (Fig. 4). This also supports the conclusion that pre-PEGIS is successfully synthesized as an unsaturated copolyester prepolymer.

**Figure 5:**
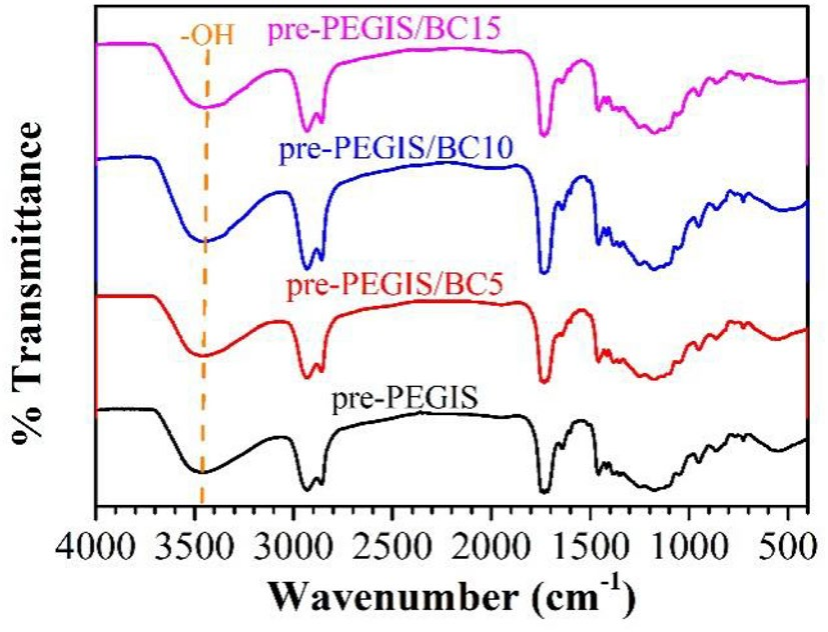
FTIR spectrum of pre-PEGIS, pre-PEGIS/BC5, pre-PEGIS/BC10, and pre-PEGIS/BC15.

The FTIR spectrum of pre-PEGIS/BC (Fig. 5) shows that there are no new peaks or disappearance of existing peaks in the polyester prepolymer after the addition of BC, indicating the absence of any chemical reactions. Since BC has abundant hydroxyl groups, and there are some residual hydroxyl groups in the polyester prepolymer, hydrogen bonding may exist. According to the Fig. 5, with the introduction of BC, the hydroxyl characteristic peaks of the polyester prepolymer gradually shift towards lower wavenumbers, demonstrating the presence of hydrogen bonding.

**Figure 6:**
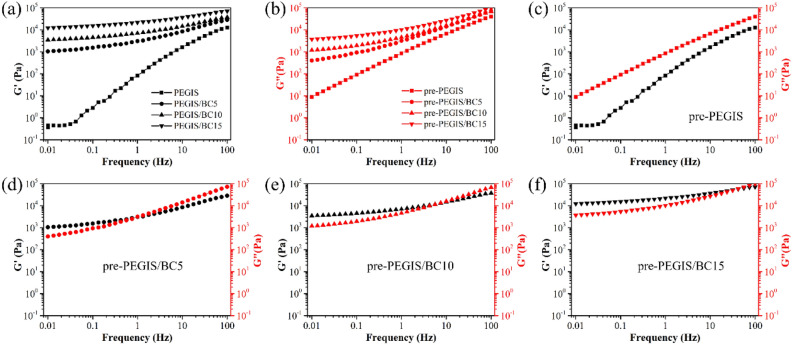
Rheological behaviors of pre-PEGIS and pre-PEGIS/BC: black line represents the storage modulus and red line represents loss modulus; (**a**) the summary of storage modulus of all four types of samples; (**b**) the summary of loss modulus; and (**c**–**f**) respectively show the rheological behaviors of pre-PEGIS, pre-PEGIS/BC5, pre-PEGIS/BC10 and pre-PEGIS/BC15.

The key point of this work is the in-situ cross-linking of PEGIS from the pre-PEGIS with photoinitiator HHMP by UV irradiation, under the framework of naturally extended bacterial cellulose. Therefore, before UV curing, it is critical to confirm that the BC dispersion is in well-extended form as we expected. Rheology of pre-PEGIS/BC has been done to testify this by observing the cross-linking degree of the materials. Since at this point, if there is some cross-linkage existing it shall be mainly contributed by the BC backbone network, and vice versa, agglomerated BC cannot present this as a hard-coiled additive. According to the analysis of the rheological behaviors of pre-PEGIS and pre-PEGIS/BC (Fig. 6), the storage modulus and loss modulus of pre-PEGIS increases continuously with the addition of BC. As shown in Fig. 6c, the loss modulus of pre-PEGIS is larger than its storage modulus, which means that pre-PEGIS is mainly a liquid of viscous deformation. And in Fig. 6e–f, the storage modulus of pre-PEGIS/BC blends are similar to their loss modulus, which proves that pre-PEGIS/BC blends are semi-solid. Furthermore, with the increase of BC, the storage modulus of pre-PEGIS/BC blends gradually exceed the loss modulus, implying the entire composite is effectively dominated by the BC backbone with a positive correlation to the amount.

**Figure 7:**
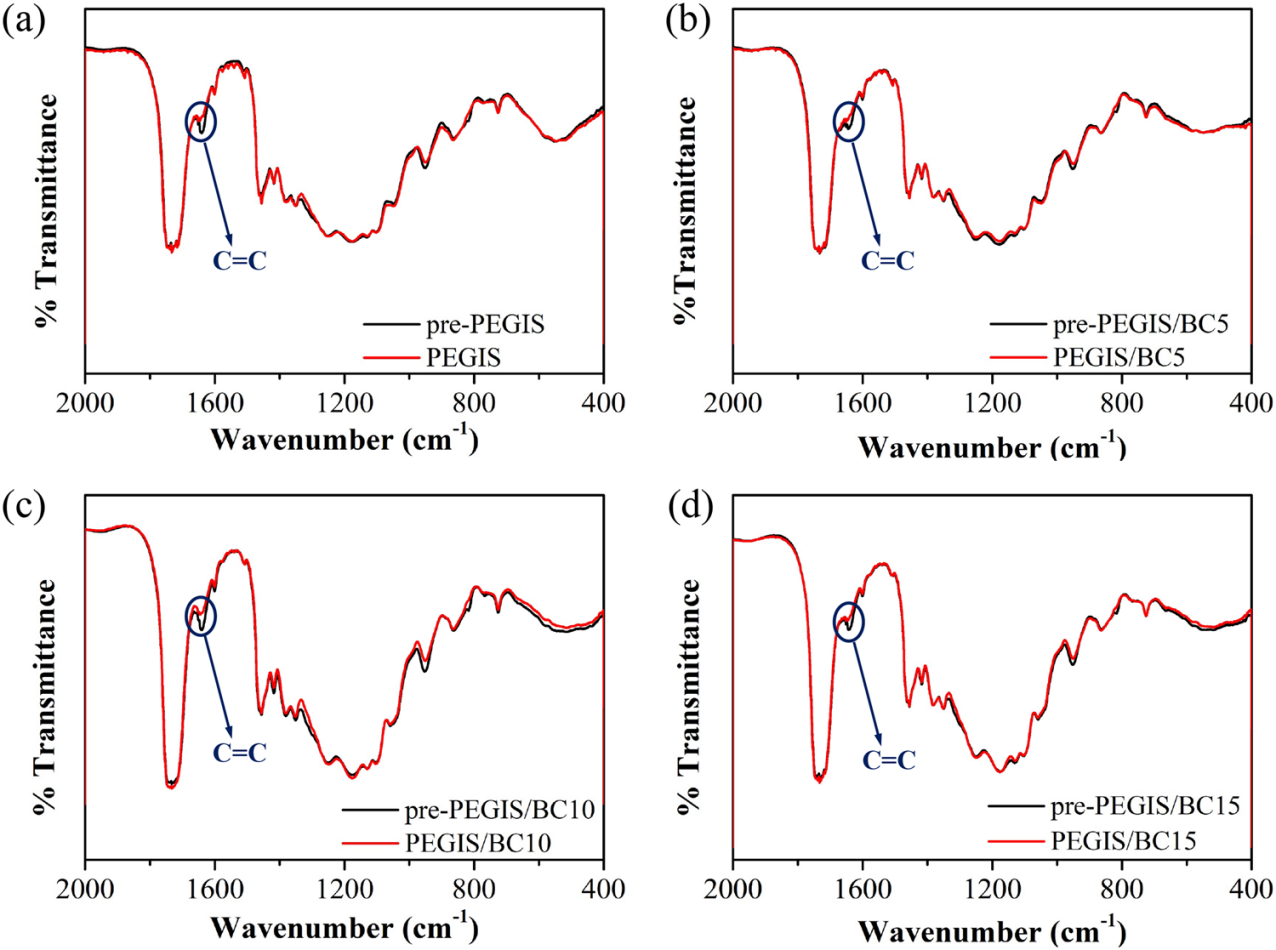
FTIR spectrums of pre-PEGIS and PEGIS before and after ultraviolet curing.

The FTIR can also help to determine the successful cross-linkage by photoinitiator and UV curing. In Fig. 7, it is exhibited that the absorption peak of carbon–carbon double bond at 1650 cm^−1^ diminished after UV curing in all the four types of pre-PEGIS/PEGIS samples: While before and after curves well overlapped in the full region, the disappear of C=C signal is quite obvious. Gel-sol content analysis is one of the methods to characterize the crosslinking degree of samples. The cross-linked part swells and the non-cross-linked part leaches out from the copolyester elastomer in THF solution. By weighing and calculating, the gel content is determined to be 62.37 ± 4.53%, 63.15 ± 3.81%, 63.90 ± 6.17% and 64.88 ± 5.35% for the PEGIS, PEGIS/BC5, PEGIS/BC10 and PEGIS/BC15. The data indicate that the degree of cross-linking increases along with the content of BC.

### Thermal and mechanical property

**Figure 8:**
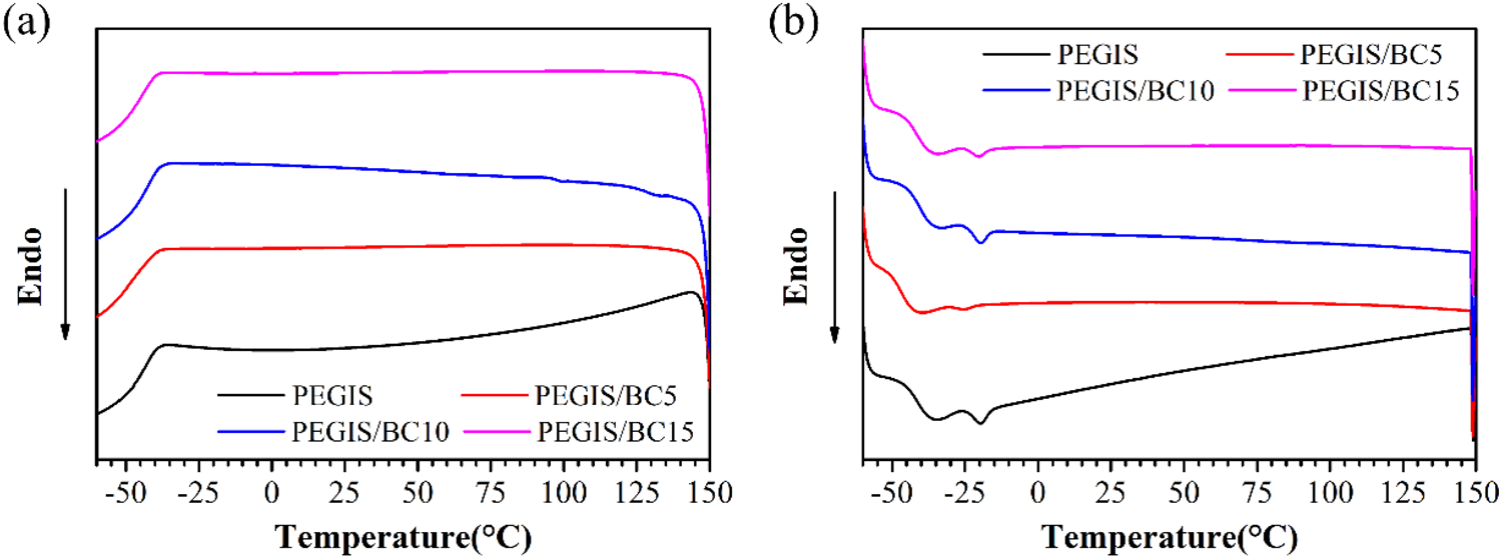
DSC cooling scan (**a**) and heating scan (**b**) of PEGIS and PEGIS/BC blends.

The DSC results of PEGIS and PEGIS/BC blends are shown in Fig. 8: in both cooling and heating process, a secondary transition at − 40 ~ − 35 °C can be located and identified as the glass transition, which is far lower than room temperature as usual rubber material as a critical characteristic to the elastomer. Furthermore, another interesting found is the minor melting peak at − 20 ~  − 15 °C in the heating curve, which does not present in cooling process. Given that ideal elastomer has no melting transition, there implies a portion of crystalized linear chains may still exist in the present material, which is expectable since the degree of cross-linking for star-polyester is more difficult to achieve than traditional vulcanized rubber. And this in another way imprints the importance of external cross-linking reinforcement such as BC network.

**Figure 9:**
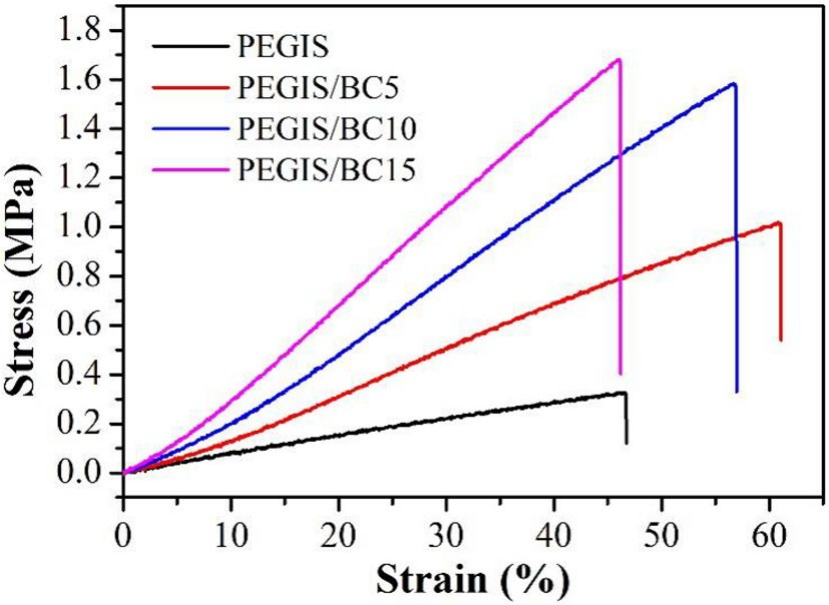
Tensile stress–strain curves of PEGIS and PEGIS/BC blends.

The mechanical properties of polyester elastomers were evaluated by tensile test, and the stress–strain curves of PEGIS and PEGIS/BC are shown in Fig. 9 and the mechanical results of PEGIS and PEGIS/BC are listed in Table 2 (Error bars are derived by conducting five replicate tests). The tensile strength of pure PEGIS is 0.33 ± 0.02 MPa, the Young's modulus is 0.90 ± 0.04 MPa, and the elongation at break is 46.5 ± 1.3%. With the addition of BC, the tensile strength, Young’s modulus and elongation at break of PEGIS/BC were significantly improved to varying degrees. And with the increase of the amount of BC, the tensile strength and Young’s modulus of PEGIS/BC also increase. The tensile strength of PEGIS/BC5, PEGIS/BC10 and PEGIS/BC15 are 1.02 ± 0.08 MPa, 1.58 ± 0.03 MPa and 1.68 ± 0.06 MPa, which are 3.09 times, 4.79 times and 5.09 times that of the pure PEGIS, respectively. The Young’s modulus of PEGIS/BC5, PEGIS/BC10 and PEGIS/BC15 are 1.15 ± 0.10 MPa, 1.86 ± 0.07 MPa and 2.44 ± 0.12 MPa, which are 1.28 times, 2.07 times and 2.71 times that of the pure PEGIS, respectively. On the other hand, it is found a slight content of BC (5 wt‰) can also improve the elongation at break to 61.0 ± 1.8%. Nevertheless, along with the higher content of BC, the increase of cross-linking degree, which plays the key role in strengthening, also leads to the decrease of elongation at break. The elongation rate of PEGIS/BC10 and PEGIS/BC15 are 56.8 ± 0.6% and 46.1 ± 1.1%, respectively.

**Table 2:** Mechanical behavior parameters of PEGIS and PEGIS/BC.

	PEGIS	PEGIS/BC5	PEGIS/BC10	PEGIS/BC15
Tensile strength (MPa)	0.33 ± 0.02	1.02 ± 0.08	1.58 ± 0.03	1.68 ± 0.06
Elongation at break (%)	46.5 ± 1.3	61.0 ± 1.8	56.8 ± 0.6	46.1 ± 1.1
Young's modulus (MPa)	0.90 ± 0.04	1.15 ± 0.10	1.86 ± 0.07	2.44 ± 0.12

### Morphology

**Figure 10:**
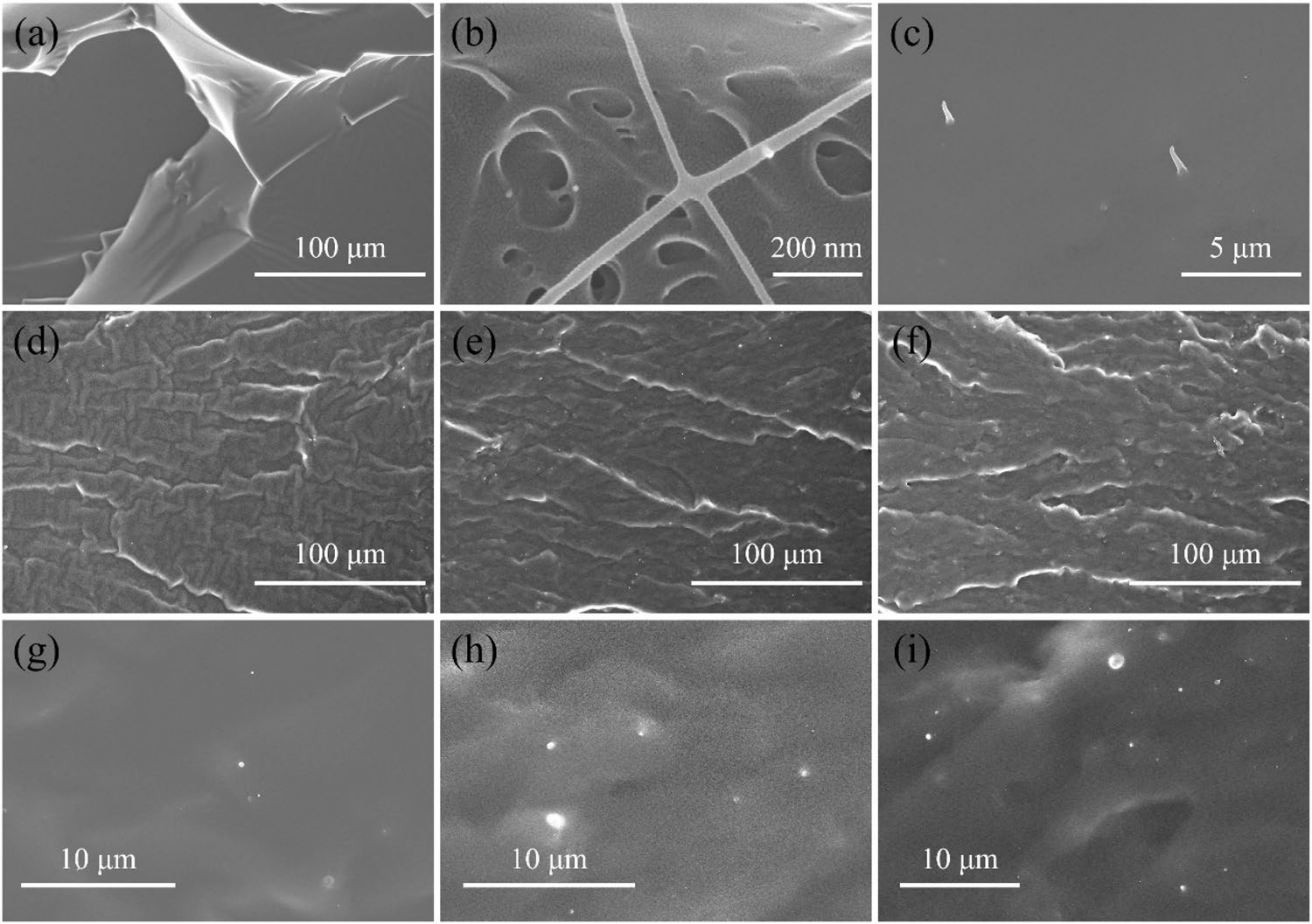
SEM image of tensile section of pure polyester PEGIS (**a**); SEM images of pure BC (**b**) and BC after being stretched in the tensile section (**c**); SEM images of tensile section of PEGIS/BC5 (**d**, **g**), PEGIS/BC10 (**e**, **h**), and PEGIS/BC15 (**f**, **i**) with different magnification.

To observe the morphology and composite state inside the elastomer, a series of SEM have been done on the surface of cross section of the samples broken in tensile test. Figure 10a is the SEM image of pure polyester PEGIS, which is an ordinary tensile broken section. For reference, the image of pure BC is shown in Fig. 10b, and as shown in Fig. 10c in the tensile section of PEGIS/BC, two break points of BC is observed, presenting obvious necking phenomenon by stretching. It reassures that BC absorbed partial energy during the tensile process, which enhanced the mechanical properties of the PEGIS/BC blends. The SEM images of tensile section of PEGIS/BC5, PEGIS/BC10, PEGIS/BC15 with different magnifications are shown in Fig. 10d–i respectively, and all present obvious existence of BC fibers or break points, once again support the successful achievement of naturally extended BC backbone network inside the PEGIS elastomer.

### Biodegradability

Biodegradability is the key advantage of the as-prepared elastomers PEGIS and PEGIS/BC blends. In composting conditions, the biodegradability of as-prepared elastomers was preliminarily compared and evaluated. The degradation rate of the samples was calculated by equation:

$${\text{D}}\% = \left( {{\text{m}}_{{\text{o}}} - {\text{ m}}_{{\text{f}}} } \right)/{\text{m}}_{{\text{o}}}$$

where D% is the degradation rate, m_o_ and m_f_ represent the initial mass and the mass after composting of the samples, respectively.

After a 30 days of compost decomposition, the pure PEGIS elastomer has entirely degraded, according to Table 3 (Error bars are derived by conducting ten replicate tests), while the PEGIS mixed with BC have not. And the rate of breakdown of elastomer slows down as more BC is applied. It is demonstrated that the addition of BC can alter the biodegradability of the PEGIS in addition to improving its strength, which is another advantage in future industrial usage to avoid the problem such as short storage period and shelf-life.

**Table 3:** Degradation rate of PEGIS and PEGIS/BC in 30 days.

Samples	Degradation (%)
PEGIS	100 ± 0
PEGIS/BC5	97.22 ± 1.13
PEGIS/BC10	59.69 ± 2.15
PEGIS/BC15	54.33 ± 3.43

## Conclusion

The biodegradable polyester elastomer PEGIS was successfully synthesized by esterification, polycondensation and UV curing. By solution blending BC can be uniformly dispersed into the polyester matrix without agglomeration to reinforce the elastomer. It was demonstrated that only a small amount of BC (15 wt‰) can significantly reinforce the elastomer: the tensile strength increased from 0.33 ± 0.02 MPa to 1.68 ± 0.06 MPa (5.09 times) and the Young's modulus increased from 0.90 ± 0.04 MPa to 2.44 ± 0.04 MPa (2.71 times), while the elongation at break is enhanced from 46.5 ± 1.3% to 61.0 ± 1.8% by only 5 wt‰ of BC content, and though this property drops down with more BC but still maintains the same with 15 wt‰ of BC content. Therefore, all three recipes we investigated here have its own advantages respectively. And in practice the selection can be adjusted to the specific requirement. It appears the PEGIS/BC10 is the optimal candidate with good compromise between mechanical performances and biodegradability. However, PEGIS/BC15 will be the suitable choice if a better modulus is required, and PEGIS/BC5 on the other hand meets much stricter requirement on the fast degradation. Overall, the efficacy of the reinforcement technique “in-situ secondary network construction” of BC, has been established, providing a pathway for the advancement of biodegradable elastomers in various future applications.

## Data Availability

The main data generated or analysed to support the conclusion during this study are included in this published article. The full datasets generated and/or analysed during the current study are available from the corresponding author by request.
